# Role of TLR4 in Neutrophil Dynamics and Functions: Contribution to Stroke Pathophysiology

**DOI:** 10.3389/fimmu.2021.757872

**Published:** 2021-10-21

**Authors:** Violeta Durán-Laforet, Carolina Peña-Martínez, Alicia García-Culebras, María Isabel Cuartero, Eng H. Lo, María Ángeles Moro, Ignacio Lizasoain

**Affiliations:** ^1^ Unidad de Investigación Neurovascular, Departamento de Farmacología y Toxicología, Facultad de Medicina, Universidad Complutense de Madrid, Instituto Universitario de Investigación en Neuroquímica, Universidad Complutense de Madrid and Instituto de Investigación Hospital 12 de Octubre (imas12), Madrid, Spain; ^2^ Neurovascular Pathophysiology Group, Centro Nacional de Investigaciones Cardiovasculares Carlos III (CNIC), Madrid, Spain; ^3^ Neuroprotection Research Laboratory, Departments of Radiology and Neurology, Massachusetts General Hospital and Harvard Medical School, Charlestown, MA, United States

**Keywords:** inflammation, myeloid, stroke, neutrophil, neuroprotection, TLR4

## Abstract

**Background and Purpose:**

The immune response subsequent to an ischemic stroke is a crucial factor in its physiopathology and outcome. It is known that TLR4 is implicated in brain damage and inflammation after stroke and that TLR4 absence induces neutrophil reprogramming toward a protective phenotype in brain ischemia, but the mechanisms remain unknown. We therefore asked how the lack of TLR4 modifies neutrophil function and their contribution to the inflammatory process.

**Methods:**

In order to assess the role of the neutrophilic TLR4 after stroke, mice that do not express TLR4 in myeloid cells (TLR4^loxP/Lyz-cre^) and its respective controls (TLR4^loxP/loxP^) were used. Focal cerebral ischemia was induced by occlusion of the middle cerebral artery and infarct size was measured by MRI. A combination of flow cytometry and confocal microscopy was used to assess different neutrophil characteristics (circadian fluctuation, cell surface markers, cell complexity) and functions (apoptosis, microglia engulfment, phagocytosis, NETosis, oxidative burst) in both genotypes.

**Results:**

As previously demonstrated, mice with TLR4 lacking-neutrophils had smaller infarct volumes than control mice. Our results show that the absence of TLR4 keeps neutrophils in a steady youth status that is dysregulated, at least in part, after an ischemic insult, preventing neutrophils from their normal circadian fluctuation. TLR4-lacking neutrophils showed a higher phagocytic activity in the basal state, they were preferentially engulfed by the microglia after stroke, and they produced less radical oxygen species (ROS) in the first stage of the inflammatory process.

**Conclusions:**

TLR4 is specifically involved in neutrophil dynamics under physiological conditions as well as in stroke-induced tissue damage. This research contributes to the idea that TLR4, especially when targeted in specific cell types, is a potential target for neuroprotective strategies.

## Introduction

Stroke is the leading cause of death and disability world-wide. It affects 13.7 million people globally per year and causes 5.5 million deaths which means that an estimated of 1 in 4 adults will experience a stroke in their lifetime (1). The immune response subsequent to an ischemic stroke is a crucial factor in stroke physiopathology and outcome. The inflammatory cascade is activated within seconds after vessel occlusion and therefore the inflammatory process starts in the intravascular compartment but, rapidly, inflammatory mediators generated *in situ* propagate through the whole organism creating a systemic response (2).

TLR4 (toll-like receptor 4) plays a key role in the immune response elicited after stroke (3). Several studies have proven that the lack of TLR4 entitles improved neurological and behavioral outcomes in different animal models of experimental stroke (4, 5). This fact has also been demonstrated in patients, in which the up-regulation of TLR4 correlates with higher inflammation and poor outcome (6). Recently, it has been shown that the administration of a TLR4-binding DNA aptamer exerts a protective effect against acute stroke in animal models (7). The fact that TLR4 is expressed in CNS as well as in circulating cells makes it harder to understand the implication of the receptor in a systemic way. Chimeric mice with bone marrow lacking TLR4 showed a reduced infarct volume and edema after experimental stroke indicating that peripheral cells have an important role in stroke pathophysiology (8). However, the specifics of the peripheric contribution of TLR4 to the ischemic process are not fully understood.

Neutrophils speedily infiltrate into the ischemic brain as part of the sterile inflammation situation caused by the ischemia. During this acute stage neutrophils contribute to BBB disruption (9), infarct size (10), hemorrhagic transformation (11) and worse neurological outcomes (12). In the last few years evidence has come up suggesting the presence of distinct neutrophil subsets in different pathologies such as infections, inflammation and cancer (13, 14). The heterogeneity of neutrophil subsets has also been studied in cardiovascular diseases (15). Whereas the detrimental effects of neutrophils in stroke pathology dominate the literature, it is being discussed whether they can also contribute to the tissue repair or even have a neuroprotective role. This is known to happen in macrophages, who are known to polarize into different functional phenotypes, such as M2, in response to microenvironmental changes (16). M2 macrophages are associated with anti-inflammatory and tissue repair functions and can be identified by the expression of certain markers such as Ym1 (also known as CHI3L3), CD206 or arginase I (17). In the stroke context, the expression of an M2 marker has been described in neutrophils. These alternative neutrophils were first described in stroke after the treatment with a PPARγ agonist, rosiglitazone (18). After stroke, infiltrated neutrophils expressed Ym1 and CD206, which increased after treatment with rosiglitazone. This treatment also produced neuroprotection and this correlated with an increased number of N2 neutrophils in the injury site (18). This reported expression of Ym1 marker in neutrophils, a bona fide marker of M2 polarization is specially remarkable since it expression in brain has previously been associated with neuroprotection (19). This neuroprotective profile has been proposed to be driven also by TLR4 absence since the lack of TLR4 likewise increased neutrophil infiltration concomitant to neuroprotection after pMCAO together with a shift of neutrophils towards an Ym1+, N2 phenotype (20, 21).

Its impact on outcome underlines the importance of the identification and characterization of myeloid cell subsets and their activation states. In addition, it is also essential to explore the signaling pathways by which these states determine stroke progression and outcome. The aim of this study is therefore to elucidate how the lack of TLR4 can modify neutrophil function and therefore alter its contribution to the inflammatory process. The comprehension of these processes may pave the way to novel therapeutic avenues to stop the cytotoxic phase of the inflammatory response and to promote neuroprotection and tissue repair after ischemic stroke.

## Materials and Methods

### Animals

Adult mice from 8 to 10 weeks were used to perform the experiments. Different genotypes of mice were used. WT mice (B6.C57BL/6J) were obtained from Harlan. To elucidate the specific role of TLR4 in different cells transgenic mice expressing the Cre recombinase enzyme under the lysozyme M promoter were used (B6.129P2-Lyz2tm1(cre)Ifo/J, Jackson Laboratory). Those animals were crossed with TLR4^loxP/loxP^ mice, kindly donated by Prof. Timothy Billiar (University of Pittsburgh, USA) to obtain mice lacking TLR4 specifically in myeloid cells (TLR4 lack of expression was routinely checked by qPCR resulting in a TLR4 expression similar to the one detected in TLR4 full knock-out mice). Mice had access to rodent chow and water ad libitum in a 12 h light/dark cycle room. All groups were performed and quantified in a randomized fashion by investigators blinded to the specific treatments. All procedures were performed in accordance with the European Parliament and of the Council Directive 2010/63/EU and Spanish legislation (RD 53/2013) and were approved by the Ethics Committee on Animal Welfare of University Complutense (PROEX number 016/18) and are reported according to ARRIVE guidelines (Animal Research: Reporting of *In Vivo* Experiments).

### Induction of Focal Ischemia

Surgery was conducted under anesthesia with isoflurane in a mix of O2 and synthetic air (0.2/0.8 L/min). Through the procedure body temperature was maintained at 37.0°C using a servo-controlled rectal probe-heating pad. Surgical procedure is a variant of the one described by Chen and collaborators (22). Firstly, the ipsilateral common carotid artery (CCA) was permanently occluded. Then, an incision is made through the middle line between the left eye and the auditive conduct. Temporal muscle is then exposed and retracted. A small craniotomy is made over the trunk of the left MCA. pMCAO was performed by ligature of the trunk just before its bifurcation between the frontal and parietal branches with a 9-0 suture (proximal occlusion) (n=10). Flow disruption was confirmed visually under an operating microscope. These experimental conditions led to moderately sized cortical infarcts. Mortality is non-existent after MCAO in this model and it is unaffected by the different experimental groups.

### Brain Infarct Determination

The infarct extension was determined by magnetic resonance imaging (MRI). MRI was performed 24 h after pMCAO using a BIOSPEC BMT 47/40 (Bruker, Ettlingen, Germany). T2-weighted images were acquired, and infarct volume was calculated using the MRI analysis calculator application from Image J software (NIH, USA) (18). To calculate the infarct volume as the percentage of the hemisphere that is infarcted, we estimated the volume of the contralateral hemisphere (CH) and that of the non-lesioned ipsilateral hemisphere (NLH) in 16 coronal sections between -1.78 and -3.64mm posterior to bregma (450 μm apart) (**Supplemental Figure 1B**, bottom). The % of the infarcted hemisphere was then calculated using the formula = (CH-NLH/CH) x 100. The value resulting from this formula is then normalized by the edema index which is the ratio between the volume of the contralateral and ipsilateral hemisphere.

### Blood Processing

Blood samples were obtained either from the tail vein, submandibular vein or directly from the right ventricle before perfusion. Blood cells were lysed in room temperature (RT) lysis buffer (50 mmol/L Tris-HCl, pH 7.4, 150 mmol/L NaCl, 5 mmol/L CaCl2, 0.02% NaN3, 1% Triton X-100).

### Flow Cytometry

Blood samples were obtained from the tail vein. All cell pellets were resuspended in FACS buffer. Cell suspensions were transferred to a 96-well plate and low affinity Fc receptors blocked by cell incubation with anti-CD16/32 antibody for 30 min. Plates were centrifuged (400 g, 5 min), supernatants discarded, and cell pellets disrupted by gentle agitation of plates. For flow cytometric analysis after MCAO or naïve animals, blood cell suspensions were added to a 96-well plate for cell surface labelling and stained with the following fluorochrome-conjugated monoclonal antibodies for 30 min: anti-mouse CD62L (clone 17A2, 1 μg/ml, BioLegend); anti-mouse/human CD11b-APC-Cy7 (clone M1/70, 1 μg/ml, BioLegend); anti-mouse CXCR4 (clone AFS98), 1 μg/ml, BioLegend); anti-mouse Ly6G (clone HK1.4, 1 μg/ml, BioLegend); anti-mouse Gr1 (clone 1A8, 1 μg/ml, BioLegend. Plates were centrifuged (400 g, 5min), supernatants discarded, and cells resuspended in FACS buffer. Data were acquired using a FACS Calibur (BD Biosciences) and analyzed using FlowJo software.

### Quantification of Neutrophil Granules

Blood samples were obtained from the submandibular vein. Blood smears were performed onto Superfrost Plus microscope slides (Thermo Scientific), air-flow dried and immediately fixed in 4% PFA for 10 min at RT. Smears were incubated with blocking solution containing 25% FBS and 0.1% Triton for 30 min and stained with anti-MPO antibody (1:200) at 4°C overnight. After washing secondary horse anti-goat biotinylated antibody (1:250) was added and smears were incubated for 2 h RT. Afterwards, smears were washed and incubated with Alexa-488 conjugated streptavidin (1:400) for 1 h at RT. Prior to mounting the smears cells were stained with DAPI (4,6-diamidino- 2-phenylindole) for 5 minutes. Imaging was performed using a Leica SP8 X confocal microscopy system coupled to a DMI6000 inverted microscope, with ×100 (HC PL Apo CS2 100×/1.4 oil) magnification objective. Granule contents were analyzed using the Spots tool (estimated XY diameter = 0.2 μm and estimated Z diameter = 0.6 μm) from Imaris software. In total, 15 neutrophils were analyzed per mouse (n=3).

### Immunofluorescence

Animals were euthanized 48 h after pMCAO by isoflurane overdose followed by transcardiac perfusion with 0.1M phosphate buffer followed by 4% paraformaldehyde (PFA) in 0.1M phosphate buffer (pH 7.4). Brains were removed, placed in 4% PFA overnight and then transferred into a 50 ml Falcon tube filled with 30% sucrose solution for 48h. Coronal series sections (40 μm) were sliced on a freezing microtome (Leica SM2000R, Leica Microsystems GmbH, Wetzlar, Germany) and stored in a cryoprotective solution. Immunofluorescence was performed on free-floating sections and incubated overnight at 4°C with the following primary antibodies: rabbit anti-mouse Ym1 (Stem Cell Tech. Inc.), Biosciences), rat anti-mouse NIMP-R14 (Abcam). Secondary antibodies used were goat anti-rabbit biotin, donkey anti-rat 647 or goat anti-rabbit biotin (Vector laboratories) in combination with Alexa 555 streptavidin (molecular probes). Sections were fixed again with 4% PFA and the same protocol was followed to stain the sections with anti-iba1 (Wako) followed by goat anti-rabbit biotin (Vector laboratories) and Alexa 488 streptavidin (Molecular probes). Controls performed in parallel without primary antibodies showed very low levels of nonspecific staining. Image acquisition was performed with a laser-scanning confocal imaging system (Zeiss LSM710) and image analysis was performed with the ZEN 2009 software (Zeiss). All co-localization images shown were confirmed by orthogonal projection of z-stack files.

### Apoptosis Assay

In order to study the apoptotic process in circulating neutrophils, blood was collected at ZT1 from tail vain (basal and 24h time points) or by cardiac puncture (48h). After blood processing, cells were resuspended at a density of 10^6^ cells/ml in FACS buffer. 0.2 ml cell suspension was pipetted into a flow cytometry test tube. 5 μl of 0.2 mM NucView 488 substrate stock solution was added to tube and mixed well to obtain a final concentration of 5 μM. Then, cells were incubated at RT for 15 minutes, protected from light. Finally, after washing cells, 200 μl FACS buffer was added to each tube for cell staining (n=3).

### Microglia Engulfment

Neutrophil engulfment by microglia was performed as previously described (18). To assess neutrophil engulfment, merged staining of Iba1 and NIMP-R14 or Iba1, NIMP-R14 and Ym1 was analyzed using a laser-scanning confocal imaging system (Zeiss LSM710). All ischemic core was photographed using the 20x magnification objective. A Z-stack of 20 μm was acquired formed of 16 Z planes (1.25 μm per plane). Quantification of double or triple labelled cells in the orthogonal projection of z-stack files was performed using the cell counter tool of the software Image J (NIH) and corroborated with the colocalization tool of the software ZEN (Zeiss) which calculates several parameters: colocalization coefficient Ch1-T1, colocalization coefficient Ch2-T2, weighted colocalization coefficient Ch1-T1, weighted colocalization coefficient Ch2-T2, overlap coefficient and correlation R. Values higher than 0.65 were considered as a colocalization which was corroborated by assessing the orthogonal projection (**Supplemental Figure 2**). In order to perform these calculations two channels were selected at a time (green *vs* red, green *vs* blue and blue *vs* red). The parameters analyzed were: 1) % of microglia/macrophages engulfing neutrophils (% of Iba1+ cells containing NIMP-R14+ particles) was calculated as the ratio between the number of Iba1+ cells engulfing NIMP-R14+ particles divided by the total number of Iba1+ cells found in the field; 2) % of cleared neutrophils (% of neutrophils engulfed by microglia/macrophages) was calculated as the ratio between the number of NIMPR14+ cells engulfed by Iba1+ cells divided by the total number of NIMP-R14+ cells found in the field; 3) % of cleared Ym1+ neutrophils (% of Ym1+ neutrophils engulfed by microglia/macrophages) was calculated as the ratio between the number of Ym1+ NIMPR14+ cells engulfed by Iba1+ cells divided by the total number of Ym1+ NIMP-R14+ cells found in the field; 4) % of cleared Ym1- neutrophils (% of Ym1- neutrophils engulfed by microglia/macrophages) was calculated as the ratio between the number of Ym1- NIMPR14+ cells engulfed by Iba1+ cells divided by the total number of Ym1- NIMP-R14+ cells found in the field (n=3).

### Neutrophil Isolation With Magnetic Beads

Neutrophil isolation was achieved by using a commercial kit by Miltenyi Biotec. Briefly, after removing RBC, cells were resuspended in 200 μl of neutrophil isolation buffer [PBS 1X, pH 7.2, 0.5% bovine serum albumin (BSA), and 2 mM ethylenediamine tetraacetic acid (EDTA)]. 50 μl of Neutrophil Biotin-Antibody Cocktail was added and cells were well mixed and incubated for 10 min in the refrigerator. 10 ml of buffer was added to wash cells and suspension was centrifuged at 300xg for 10 minutes. Pellet was resuspended in 400 μl of buffer and 100 μl of Anti-Biotin MicroBeads were added. Cell suspension was well mixed, and cells were incubated for 15 minutes in the refrigerator. After incubation, cells were washed with 10 ml of buffer and centrifuged at 300xg for 10 minutes. Cell pellet was resuspended in 500 μl of buffer. The column was placed in the magnetic field (MACS Separator) and prepared by rinsing with 500 μl of buffer. Cell suspension was applied onto the column. Flow-through was collected, representing the enriched neutrophils. Then, column was washed three times and flow-through combined with the one obtained in the previous step.

### NET Formation Assay

Blood was collected at ZT1 by cardiac puncture. After neutrophil and isolation, 1x10^6^ cells were resuspended in RPMI. Neutrophils were then added to an Ibidi chamber (μ-Slide 8 Well Glass Bottom). Neutrophils were incubated to 15 min at 37°C so that cells adhere to the bottom of the chamber. Phorbol 12-myristate 13-acetate (PMA, 20 nmol/L in RPMI) was added and cells are incubated at 37°C for 3h. After incubation, supernatant was removed and 4% PFA is added in order to fix the cells (10 min RT). After fixation, cells were washed and stained for NIMP-R14, elastase and citrunillated histone H3 (H3Cit) markers (rat anti-NIMP 1:200, rabbit anti-elastase 1:300, rabbit anti-H3Cit 1:400). Confocal images were taken in order to quantify the percentage of formed NETs. NETs quantification was performed by counting the total number of neutrophils (NIMP-R14+ cells) per well and the number of neutrophils undergoing NETosis (NIMP-R14+/H3Cit+/Elastase+ cells). Cells expressing H3Cit and elastase but no NIMP-R14 were also quantified as of NET-like events. Results are expressed as percentage of neutrophils that are undergoing NETosis or are NET-like events (n=6).

### Phagocytic Activity of Myeloid Cells

Blood was collected by cardiac puncture at ZT1 into a 15 ml Falcon tube previously filled with 10% citrate solution. RBC were lysed with 5 ml RBC lysis buffer (7 min) and blood centrifuged for 5 min at 1800 revolutions per minute (rpm). Supernatant was discarded and pellet resuspended in 5 ml Rosewell Park Memorial Institute (RPMI) buffer after phosphate buffer solution (PBS) 1x washing. Beads (latex beads carboxylate-modified polystyrene 1 um) were added to a final dilution of 1:100 and cell suspension was incubated for 1 h at 37°C. After incubation, cells were washed 3 times with PBS 1x, resuspended in FACS buffer and stained for surface markers (n=4-5).

### Neutrophil Oxidative Burst Assay

Blood was collected transcardially (ZT1) and placed in a 15 ml Falcon tube previously filled with 10% citrate solution. After removing RBC, WBC were washed with 300 μl of DMEM (+ glucose – phenol red) and divided in 100 ul aliquots. 1 μM of DHR 123 and 1 μM of W-peptide (when required) were added and cells were incubated for 20 min at 37°C. During that time dihydrorhodamine 123 (DHR123) is transformed into rhodamine which is a fluorescent compound and therefore detectable with flow cytometry (**Figure 5**). After incubation, cells were placed in an ice bath for 10 minutes to stop the reaction. Cells were centrifuged at 1800 rpm, 4°C for 5 min and cell pellet resuspended in 300 μl of ice-cold HBSS. Cells were then stained for flow cytometry analysis (n=4-6).

### Statistical Analysis

Results are expressed as mean ± SEM for the indicated number of experiments. Statistical significance was determined by t-test or one- or two-way ANOVA. For the latter Tukey or Bonferroni *post hoc* tests were used. Values of p<0.05 were considered statistically significant. All statistical analysis were performed with GraphPad Prism software (GraphPad Software, Inc).

## Results

In order to assess the role of TLR4 on neutrophil function after stroke, TLR4^loxP/loxP^ and TLR4^loxP/Lyz-cre^ mice were subjected to pMCAO. As previously reported, TLR4^loxP/Lyz-cre^ mice showed a reduced infarct volume determined by magnetic resonance imaging 24 h after the surgery (**Supplemental Figure 1A**, P < 0.05). Given that the absence of TLR4 in neutrophils affords neuroprotection, we decided to explore the mechanisms involved.

### Characterization of TLR4-Deficient Neutrophil Dynamics

**Figure 1:**
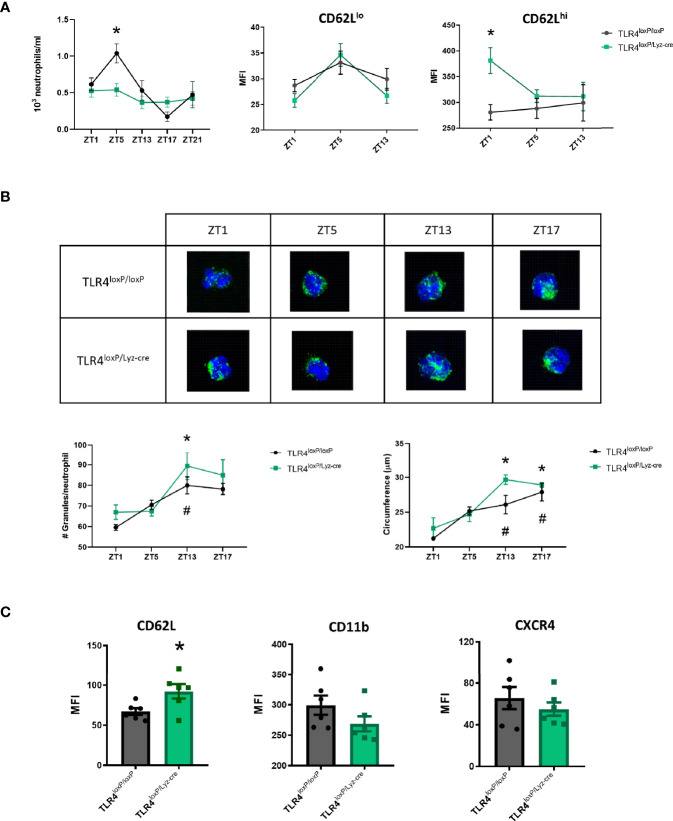
Characterization of TLR4-deficient neutrophil dynamics. **(A)** Left: Total number of neutrophils in blood at ZT1, ZT5, ZT13, ZT17, ZT21 in TLR4^loxP/loxP^ mice and TLR4^loxP/Lyz-cre^ mice. Two-way ANOVA analysis showed a significative difference in the variable time between genotypes (P<0.05, F[4, 30]=4.681, n=3-6). Bonferroni post-hoc analysis showed that at ZT5 there were less neutrophils in blood in TLR4^loxP/Lyz-cre^ mice than in TLR4^loxP/loxP^ mice (*P < 0.05 *vs* TLR4^loxP/loxP^). Middle and right: CD62L expression in neutrophils through time. CD62L MFI (mean fluorescence intensity) in CD62L^lo^ neutrophils and CD62L^hi^ neutrophils (P < 0.05, n=6). Two-way ANOVA analysis showed a trend to the difference in the genotype variable (P=0.07, F[1, 10]=4.016, n=6). Bonferroni post-hoc analysis showed a significative higher expression of CD62L at ZT1 (*P < 0.05 *vs* TLR4^loxP/loxP^, n=6). **(B)** Quantification of primary granules from confocal images. Representative images of granule content of neutrophils (green: MPO, blue: DAPI). Bottom left: number of granules per neutrophil at different time points (n=3, 15 neutrophils per mouse, *P < 0.05 *vs* TLR4^loxP/loxP^ ZT1, ^#^P < 0.05 *vs* TLR4^loxP/Lyz-cre^ ZT1). Bottom right: Neutrophil circumference (µm) (n=3, 15 neutrophils per mice, *P < 0.05 *vs* TLR4^loxP/loxP^ ZT1, ^#^P < 0.05 *vs* TLR4^loxP/Lyz-cre^ ZT1). **(C)** Neutrophil phenotype according to the neutrophil aging markers. Flow cytometry analysis of the mean fluorescence intensity of CD62L, CD11b and CXCR4 in neutrophils in the basal state at ZT1 (*P < 0.05; n=6). Data are mean ± SEM.

Neutrophil function is influenced by a circadian clock. Since previous studies from our laboratory (21) indicated that TLR4 lacking-neutrophils were similar to non-aged neutrophils (23), we assessed if the absence of TLR4 was affecting circadian fluctuations by quantifying the absolute neutrophil count in blood at different time points (Zeitgeber Time (ZT, which means one hour after lights have been turned on) 1, ZT5, ZT13, ZT17, ZT21). Our results show that TLR4^loxP/loxP^ mice exhibit a circadian pattern that was however altered in TLR4^loxP/Lyz-cre^ mice at ZT5 (**Figure 1A**, P<0.05), suggesting that the absence of TLR4 affects function by preventing neutrophils from their normal circadian fluctuation. Regarding the circadian expression of aging markers, whereas almost all neutrophils have been reported to be CD62L^hi^ at ZT13, at ZT5 there is a mixture of CD62L^hi^ and CD62L^lo^ neutrophils (24). We therefore explored the proportion of CD62L^hi/lo^ neutrophils in the absence of TLR4. First, CD62^lo^ expression was found to match with the typical circadian pattern in both genotypes. However, while TLR4^loxP/loxP^ CD62L^hi^ neutrophils did not fluctuate as expected, neutrophils lacking TLR4 had a significative higher expression of CD62L at ZT1 (**Figure 1A**, right, P<0.05).

Granularity and size are two intrinsic cell features that may influence neutrophil function, vary with a circadian pattern and also differ according to the phenotype. These characteristics can be quantified by confocal microscopy. The number of granules as well as the cell size (circumference) increased through time (**Figure 1B**, P<0.05) according to the previous literature (25) but no differences between genotypes were found.

Since the observed increase in the expression of CD62L marker in the CD62L^hi^ neutrophil population at ZT1 could indicate that they exhibit a “young” profile we decided to analyze three neutrophil aging markers (23), namely CD62L, CXCR4 and CD11b, at the same time-point. Neutrophils without TLR4 showed a higher expression of CD62L; we could also observe a trend towards a decrease of CD11b and CXCR4 expression (**Figure 1C**, P<0.05). These results suggest that, in homeostasis, the absence of TLR4 keeps the neutrophils in a steady “youth” status characterized by a higher expression of CD62L, in agreement with an anti-inflammatory phenotype previously described (23).

### Pro-Resolving Mechanisms

#### Neutrophil Apoptosis

**Figure 2:**
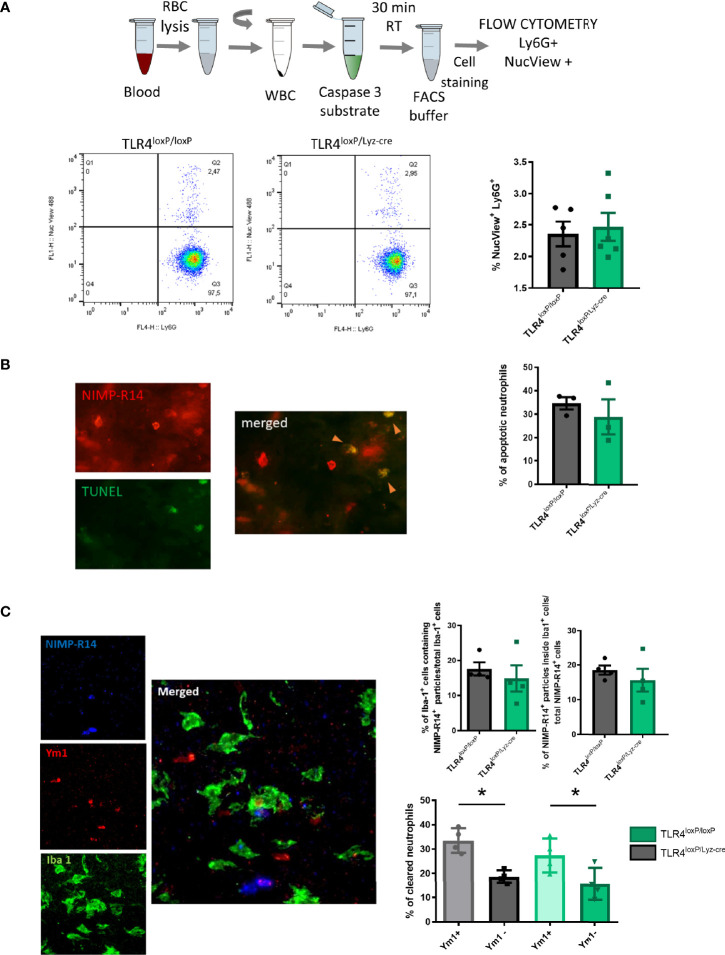
Neutrophil apoptosis and microglia engulfment. **(A)** Top: Experimental design: blood was collected, RBC lysed, WBC resuspended and finally caspase 3 substrate was added and incubated with the WBC for 30 min at RT. After incubation, cells were stained for Ly6G marker and samples were analyzed with a flow cytometer. Bottom: Flow cytometry analysis of the percentage of apoptotic neutrophils in blood (NucView^+^/Ly6G^+^). **(B)** TUNEL assay was performed in fixed brain slices and after they were stained with a neutrophil marker (NIMP-R14). Total number of neutrophils [NIMP-R14^+^ cells (top left)] was quantified. Double positive cells (NIMP-R14^+^/TUNEL^+^ cells, right) were quantified as apoptotic neutrophils (orange arrowheads). Percentage of apoptotic neutrophils infiltrated in the ischemic tissue from TLR4^loxP/loxP^ mice and TLR4^loxP/LyzM-cre^ mice (n=3). **(C)** Preferential phagocytosis of N2 neutrophils by microglia. Left: Representative phagocytosis micrograph showing neutrophil staining (NIMP-R14^+^ cells, blue), N2 marker (Ym1^+^ cells, red) and microglia (Iba1^+^ cells, green) and all merged channels showing an N2 neutrophil engulfed by microglia. Right top: Percentage of phagocytic microglia in TLR4^loxP/loxP^ mice and in TLR4^loxP/Lyz-cre^ mice (n=4) and percentage of neutrophils engulfed by microglia normalized by the total number of neutrophils (n=3). Right bottom: Quantification of clearance of specific neutrophil population (Ym1+/Ym1-) (*P < 0.05, n=4). Data are mean ± SEM.

Neutrophils have granules filled with a potentially dangerous arsenal of cytotoxic molecules (26). This fact makes apoptosis, which by definition is a controlled and programmed cell death, a key component of the pro-resolutive process. We therefore measured neutrophil apoptosis in blood neutrophils, using flow cytometry with a caspase 3 fluorescent substrate (**Figure 2A**, top), in order to assess whether clearance was associated to this process. Our results showed no differences in apoptosis in neutrophils from TLR4^loxP/loxP^ mice and TLR4^loxP/Lyz-cre^ mice (**Figure 2A**, bottom). Post-ischemic analysis of apoptosis (24 and 48 h after the stroke) neither showed any differences in apoptosis between genotypes. Two-way ANOVA analysis did reveal an increase in apoptotic neutrophils along time in both genotypes (**Supplemental Figure 3**).

We also evaluated the apoptosis of infiltrated neutrophils by performing a TUNEL assay in fixed brain slices obtained 48 h after stroke. Again, we failed to observe any differences in the percentage of apoptotic neutrophils between genotypes (**Figure 2B**). Our results therefore suggest that the presence of TLR4 does not seem to influence the intrinsic neutrophilic apoptotic process.

#### Neutrophil Engulfment by Microglia

Given their noxious potential, neutrophils are promptly removed from the inflamed tissue in order to decrease the possible damage associated with their inflammatory activity. In the brain, microglia are the responsible for that clearance. To determine the effect of neutrophil TLR4 in their susceptibility to undergo phagocytosis, we measured neutrophil clearance by microglia engulfment with simultaneous visualization of Iba1 (microglia), NIMP-R14 (neutrophil), and Ym1 (N2 or alternative neutrophil marker) in infarct cores of TLR4^loxP/loxP^ and TLR4^loxP/Lyz-cre^ mice. Confocal images showed that the cytoplasm in microglia contains NIMP-R14^+^ particles, without difference between genotypes. Moreover, the percentage of neutrophils engulfed by the microglia was the same in both genotypes (**Figure 2C**, top). Importantly, we found that microglia preferentially engulfed Ym1+ neutrophils at the ischemic core when compared with the Ym1- neutrophil subpopulation (**Figure 2C**, bottom) in accordance with previous results from our laboratory (18).

#### Neutrophil Phagocytosis

**Figure 3:**
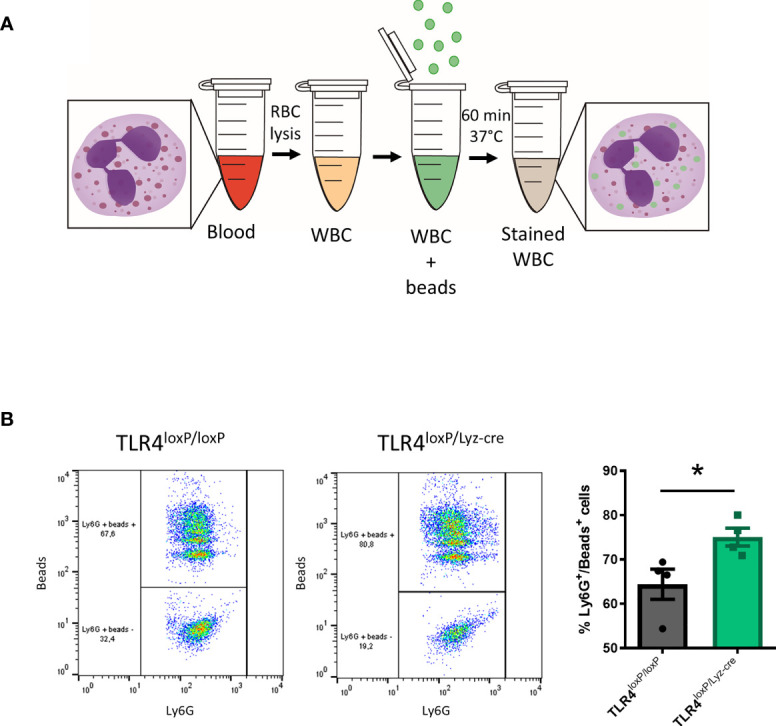
Neutrophil phagocytosis. **(A)** Experimental design of phagocytic activity of myeloid cells assay. Blood is collected, RBC are lysed, and WBC incubated for 60 min at 37°C with the fluorescent beads. After incubation cells are washed and stained for Ly6G marker. **(B)** Flow cytometry analysis of the percentage of neutrophils that had engulfed beads in TLR4^loxP/loxP^ mice and TLR4^loxP/Lyz-cre^ mice (*P < 0.05 *vs* TLR4^loxP/loxP^; n=4-5). Data are mean ± SEM.

Neutrophils are professional phagocytes. They are endowed with the capacity to engulf and thereby eliminate pathogens or cell debris. We therefore evaluated the effect of the TLR4 absence on the phagocytic activity of blood neutrophils (**Figure 3A**). Our results show that neutrophils lacking TLR4 display an increased phagocytic activity (**Figure 3B**, P<0.05). These results suggest that, in homeostasis, the lack of TLR4 renders neutrophils with a higher phagocytic activity. Also, two-way ANOVA analysis revealed a significant effect of the time [P<0.05, F(2, 19)=12.92], and Bonferroni post-hoc test showed that the phagocytic activity of neutrophils from TLR4^loxP/Lyz-cre^ mice decreases significantly over time (**Supplemental Figure 4**, P<0.05).

### Pro-Inflammatory Effector Mechanisms

#### Neutrophil Extracellular Trap (NET) Formation

**Figure 4:**
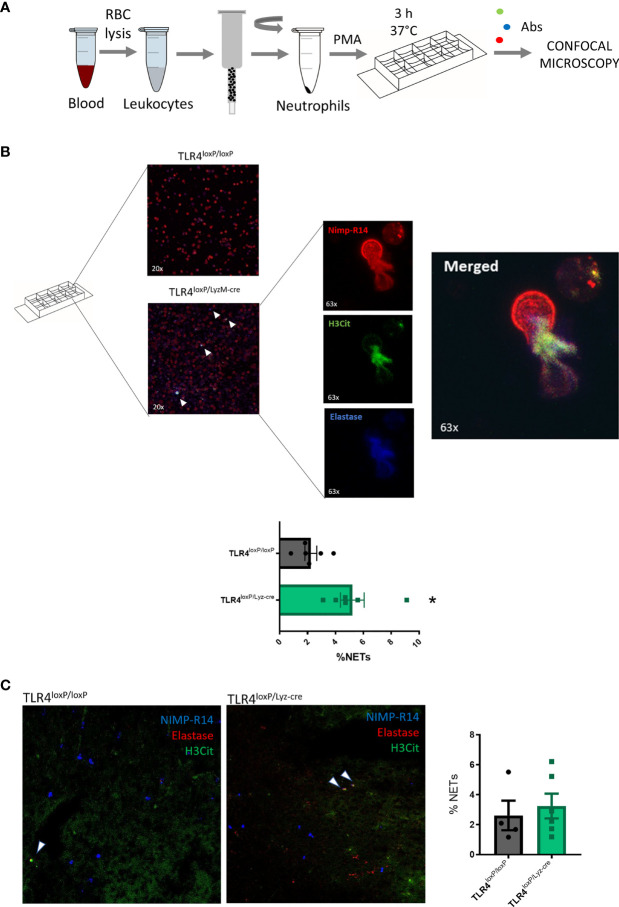
Neutrophil Extracellular Trap formation. **(A)** Experimental design: blood was collected, RBC lysed, and neutrophils sorted and stimulated with PMA. Samples were incubated for 3 h at 37°C. After incubation time, cells were washed, fixed and stained for NIMP-R14, H3Cit and Elastase. **(B)** Micrographs of neutrophils treated with PMA from TLR4^loxP/loxP^ and TLR4^loxP/Lyz-cre^ mice. NETs are marked with white arrowheads. 63x zoomed images show a neutrophil extruding its content releasing the NET. **(C)** Percentage of NETs (NIMP-R14^+^/H3Cit^+^/Elastase^+^) from TLR4^loxP/loxP^ and TLR4^loxP/Lyz-cre^ mice (*P < 0.05 *vs* TLR4^loxP/loxP^; n=6). Data are mean ± SEM.

Upon activation, neutrophils can release extracellular trap and form NETs. We wondered whether the absence of TLR4 had any effect in the ability of neutrophils to undergo NETosis. Firstly, *in vitro* (**Figure 4A**), we found that neutrophils obtained in basal state from TLR4^loxP/Lyz-cre^ were more prone to form NETs than those from TLR4^loxP/loxP^ mice (**Figure 4B**, P<0.05). In order to see if this phenomenon was also occurring *in vivo* in the ischemic tissue, we stained fixed brain slices for the same markers as before. In this case, we did not observe any differences in the percentage of NETs in the ischemic core between both genotypes at the time studied (**Figure 4C**). While analyzing the images we observed that it was common to see elastase and H3Cit signal colocalizing without any trace of NIMP-R14. This is likely due to the dispersion of the neutrophil membrane when it is disrupted after the NET release. We also quantified these NET-like events but, again, we did not detect any differences between genotypes (**Supplemental Figure 5**).

#### Reactive Oxygen Species (ROS) Production

**Figure 5:**
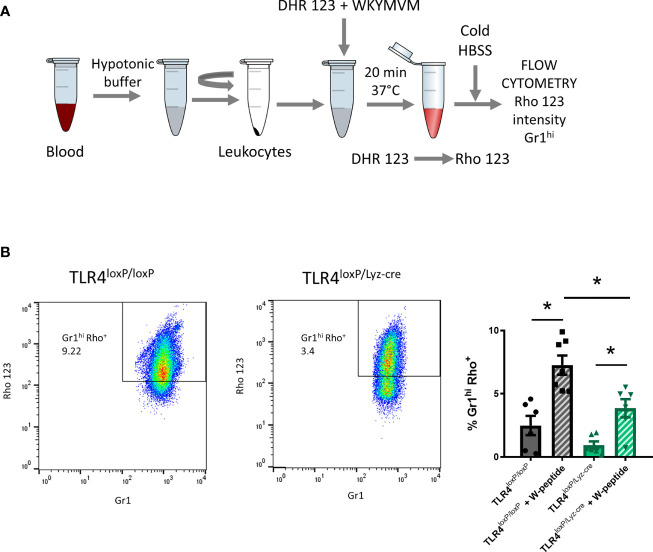
Reactive oxygen species production. **(A)** Experimental design: blood was collected, RBC lysed, WBC resuspended and DHR123 and W-peptide (if necessary) were added. Samples were incubated for 20 min at 37°C. After incubation time, cold HBSS was added to stop the reaction. Cells were stained for Gr1 marker and washed. Samples were analyzed with the flow cytometer. **(B)** Flow cytometry analysis of the percentage of neutrophils producing ROS (% Gr1^hi^Rho^+^ cells) of viable cells with and without W-peptide activation (*P < 0.05, n=4-6). Data are mean ± SEM.

The production of ROS is a key functional response of granulocytes. It is crucial when contributing to host defense, but it can also result in collateral damage of tissues in sterile inflammation. ROS production was quantified by flow cytometry by gating Gr1^hi^ and Rho^+^ cells (**Figure 5A**). We observed that, in blood neutrophils, there was a significant decrease in ROS production between TLR4^loxP/loxP^ and TLR4^loxP/LyzM-cre^ neutrophils. Also, there was a significant difference in W-peptide-induced neutrophil activation between genotypes (**Figure 5B**). At 24 and 48 h we did not longer see a difference in ROS production between genotypes (**Supplemental Figure 6A**). Two-way ANOVA analysis of activated and non-activated neutrophils of both genotypes showed a significant effect of time. Also, in the activated groups, we observed differences between genotypes in the basal state (**Supplemental Figure 6B**). Our data suggest that, after stroke, there is an increase in the production of ROS, which is decreased in neutrophils lacking TLR4, an effect which could mitigate that damage and therefore contribute to neuroprotection.

## Discussion

We among others have been long dedicated to deciphering the role of TLR4 in the pathophysiology of stroke. However, the specific mechanisms by which TLR4 influence the ischemic process and, in particular, the neutrophil TLR4, are yet to be elucidated. In this study we show that the absence of TLR4 modifies the neutrophil dynamics and alter their ability to phagocyte and to produces ROS, effects that could contribute to the neuroprotection after stroke.

First, we wanted to elucidate if the absence of TLR4 could alter the neutrophil dynamics, so we performed a thorough characterization of neutrophil features in the steady state. Neutrophils follow a circadian rhythm, with the number of total neutrophils varying during the day which has an effect in their function and phenotype (24). We wondered if the absence of TLR4 affected the regulation of the neutrophil circadian pattern. The analysis of the neutrophil kinetics in blood showed that neutrophils with TLR4 exhibited a typical circadian pattern. However, this motif was absent in TLR4-lacking neutrophils, for which their number did not oscillate through the day. Also, the expression of CD62L in CD62L^hi^ neutrophil was higher indicating that the absence of TLR4 abrogates the circadian pattern displayed by neutrophils under physiological conditions.

Our previous results showed that neutrophils without TLR4 exhibit a transcriptional profile similar to the non-aged neutrophils described by Frenette and cols (23). Now, we took a step further into characterizing these neutrophils by analyzing the expression of certain surface markers associated with non-aged neutrophil populations. Our results show that TLR4 lacking neutrophils have a higher expression of CD62L and a trend to a decrease in CD11b and CXCR4, showing a younger profile which has proven to be less inflammatory since neutrophils become more active as they age (23). Moreover, we have observed that, after the stroke, this surface marker pattern is no longer observable. This suggests that the inflammatory response subsequent to the ischemia is strong enough to dysregulate the surface marker expression process and, very likely, to interfere with the polarization process.

As previously stated, the absence of TLR4 mediates a neuroprotective effect due to the inhibition of the inflammatory response after stroke (4, 5, 27). Classically, it has been described that neutrophils are in part responsible for the ischemic damage after stroke (28–30). However, we have recently described that TLR4 absence increases the levels of alternative neutrophils (N2), an effect associated with neuroprotection after stroke (21). Considering that TLR4-lacking neutrophils are, at least, partially responsible for the neuroprotection observed in TLR4^loxP/Lyz-cre^ mice, we decided to globally assess the functionality of the TLR4-lacking neutrophil, and how this affected the outcome after stroke. In order to do so, we studied several parameters associated with neutrophil function, some of them related to their effector pro-inflammatory actions (such as ROS production and NETosis) and some other related to pro-resolving properties (such as phagocytic activity, apoptosis, microglia engulfment). Also, we asked whether those functions were in any way altered by the ischemic insult, for which we studied them in the basal state and after the stroke. A potential limitation of this study is that blood was drawn from different anatomical locations depending on the amount of blood needed for each according to animal welfare guidelines. As previously shown, the site of blood collection can influence the results (31). Further studies are required to elucidate the robustness of the results across different types of samples. Another potential limitation is that, since it has been previously shown in other publications (21) as well as replicated in this paper (**Supplemental Figure 1**) that the infarct volume decreases with the lack of neutrophil TLR4, we did not measured the infarct volume of all experimental mice. Regarding the clearance of neutrophils, which is crucial to limit the potential damage they can produce in the tissue, we investigated both apoptosis and neutrophil clearance by the microglia. We did not observe any differences between genotypes in apoptosis. Although preexisting literature established that TLR4 stimulation induces an increase in the lifespan of the neutrophils (32, 33), all prior studies were conducted by administering an external stimulus such as LPS, which is much stronger than the weak TLR4 activation that can occur in a basal state (probably just given through the microbiome). Also, the increase in neutrophil survival may be an artifact resulting from the presence of monocytes demonstrated in some highly purified neutrophil preparations, where it has been shown that variations in apoptosis rates after TLR4 activation were due to monocyte contamination (33). In order to explore the process as close to the *in vivo* situation as possible, all our assays were performed with WBC, containing monocytes that express TLR4 (at least, the majority of them), which could explain the absence of differences. Together with the observation that there is no difference in apoptotic infiltrated neutrophils we could suggest that neutrophil TLR4 does not influence the apoptotic process. Previous studies have stablished that N2 neutrophils are preferentially engulfed by microglia (18) and that the absence of TLR4 skews neutrophils to an N2 phenotype (21). We observed that N2 neutrophils are preferentially engulfed in both genotypes. Considering that TLR4^loxP/Lyz-M^ mice have a higher proportion of N2 neutrophils, that dying neutrophils ultimately disintegrate potentially liberating their cargo and contributing to the tissue damage, and that phagocytosis promotes secretion of anti-inflammatory mediators, an increase in N2 neutrophils engulfment could contribute to the resolution of inflammation.

In addition, neutrophils can have a role in the resolution of the inflammatory process since, as professional phagocytes, they can eliminate cell debris. In the basal state we observed a higher percentage of phagocytosing neutrophils in the TLR4-lacking genotype, which may contribute to their pro-resolving properties in the very early stages of the inflammatory process limiting the expansion of DAMPs due to the cell debris elimination.

One of the main functions of the neutrophil is its ability to form NETs. Surprisingly, we found that neutrophils without TLR4 are more prone to undergo NETosis *in vitro*. It has been reported that NET formation increases in aged neutrophils when stimulated with LPS (23). Also, in lung injury, NETs markers were significantly lower in TLR4KO mice than in WT mice (34). However, very recently, a new study has proposed that Bmal1^ΔN^ mice (Bmal is only lacking in neutrophils, which display a constitutive young state) show a higher proportion of PMA-induced NET-forming cells than CXCR4^ΔN^ ones (where CXCR4 is only lacking in neutrophils, which present a constitutive aged state) (25), in agreement with our results. As this process raised some questions, we examined NET formation in brain, but we did not observe any differences between genotypes. We have previously seen that neutrophils lacking TLR4 have an increased phagocytic activity in the basal state. Neutrophils that have engaged in a phagocytic process have its MPO and NE content sequestered within the phagolysosome and, therefore, are less able to reach the cytoskeleton and the nucleus, a critical checkpoint in NET formation (35, 36). Also, as we stated previously, in our genetic model platelets express TLR4 and, therefore, may play a role in triggering neutrophils activation. On their turn, it has been reported that neutrophils that have phagocytosed activated platelets are driven to an unresponsive state, unable to respond to further inflammatory stimuli failing to generate NETs (37). In summary, the NETosis process seems unlikely to be involved in the neuroprotective effect that neutrophils without TLR4 show in stroke, but we could hypothesize that reduced NETosis due to previous phagocytic activity might mitigate this damaging function.

As previously stated, neutrophils are activated by DAMPs whereby they produce ROS, which could produce additional tissue damage in a sterile inflammation context (38). We observed that neutrophils without TLR4 obtained prior to the surgery produce significantly less ROS, in agreement with the reported involvement of TLR4 in eliciting oxidative burst in neutrophils when induced by LPS (39). In contrast, 24 h after stroke, W-peptide stimulation failed to increase ROS production. Frenette and cols. described that aged neutrophils exhibited significantly increased ROS production (23) which correlates with our hypothesis. This mechanism has also been studied in myocardial infarction, where neutrophils treated with metoprolol are less capable of producing ROS, which contributes to the protective effect (40). Overall, the lack of TLR4 in neutrophils could mitigate the damage caused by neutrophils and contribute to neuroprotection, since their ability to produce ROS is decreased in the early stages of the inflammatory process.

Previous publications have shown that targeting TLR4 could have therapeutic potential. An aptamer against TLR4 has proved to be useful after acute stroke in animal models (7). Of note, a cell-specific approach may reduce adverse effects that could occur after TLR4 antagonist administration such as reduced neuronal survival and proliferation or the inhibition of phagocytosis by microglia, which is of extreme relevance in stroke pathology (41). By selectively targeting neutrophil TLR4, it would be possible to block the damaging activity of neutrophils without interfering with the other beneficial roles of TLR4 signaling after stroke. In conclusion, using loss-of-function approaches, we demonstrated that neutrophil TLR4 is involved in neutrophil dynamics under physiological conditions as well as in stroke-induced brain damage. In addition, we showed that the lack of TLR4 in neutrophils increases their phagocytic activity and limits ROS production. Taken together, these findings support a pro-inflammatory role of the neutrophil TLR4 receptor, and suggest that specifically targeting TLR4 in neutrophils may offer a novel therapeutic approach for stroke. A better characterization of the myeloid cell subsets that orchestrate the response to brain ischemia and their dynamics may help us to design specific treatments within specific therapeutic windows with the aim to increase the benefits of the modulation of cell-cell interactions in the immune response after stroke.

## Data Availability Statement

The raw data supporting the conclusions of this article will be made available by the authors, without undue reservation.

## Ethics Statement

The animal study was reviewed and approved by Ethics Committee on Animal Welfare of University Complutense (PROEX number 016/18).

## Author Contributions

VD-L, AG-C, CP-M, MM, and IL designed the research studies. VD-L, AG-C, CP-M, and MC conducted the experiments and/or acquired the data. VD-L, AG-C, CP-M, MC, MM, and IL contributed to the analysis and/or interpretation of the results. VD-L, MM, and IL wrote the manuscript. All authors contributed to the article and approved the submitted version.

## Funding

This work was supported by grants from Instituto de Salud Carlos III and co-financed by the European Development Regional Fund “A Way to Achieve Europe” PI20/00535 and RETICS RD16/0019/0009 (IL), from Regional Madrid Government B2017/BMD- 3688 (IL), from Spanish Ministry of Science and Innovation PID2019-106581RB-I00 (MM), from Leducq Foundation for Cardiovascular Research TNE-19CVD01 (MM), from Fundación La Caixa HR17_00527 (MM). The CNIC is supported by the Instituto de Salud Carlos III (ISCIII), the Ministerio de Ciencia e Innovación (MCIN) and the Pro CNIC Foundation.

## Conflict of Interest

The authors declare that the research was conducted in the absence of any commercial or financial relationships that could be construed as a potential conflict of interest.

## Publisher’s Note

All claims expressed in this article are solely those of the authors and do not necessarily represent those of their affiliated organizations, or those of the publisher, the editors and the reviewers. Any product that may be evaluated in this article, or claim that may be made by its manufacturer, is not guaranteed or endorsed by the publisher.

## Supplementary Material

Supplementary Figure 1 Effect of specific myeloid TLR4 ablation on infarct volume. **(A)** TLR4^loxP/loxP^ and TLR4^loxP/Lyz-cre^ mice were subjected to MCAO; infarct volumes were determined by magnetic resonance imaging (MRI) 24 h after ischemic insult in both groups (n=10; *P < 0.05 *vs* TLR4^loxP/loxP^). **(B)** Representative images of MRI from both genotypes (top). Schematic representation of the measured areas from MRI images (Magenta: ipsilateral hemisphere; Yellow: contralateral hemisphere; Red: infarct). Data are mean ± SEM.

Supplementary Figure 2 Microglia engulfment colocalization analysis. **(A)** Representative images used for the colocalization analysis of the different channels two by two (green vs red, green vs blue and blue vs red). **(B)** Table containing the different values obtained with the colocalization analysis tool (Zen software, Zeiss). **(C)** Orthogonal projection of the representative microglia cell used to calculate the colocalization parameters (Green: Iba1, blue: NIMP-R14, red: Ym1).

Supplementary Figure 3 Apoptosis assay in blood neutrophils. Two-way ANOVA analysis of the data showing a significative effect of time in both genotypes (Time: P<0.05, F[2, 10] 6.536; (*P < 0.05 *vs* TLR4^loxP/loxP^ pre-surgery; #P < 0.05 *vs* TLR4^loxP/Lyz-cre^ pre-surgery; $P < 0.05 *vs* TLR4^loxP/Lyz-cre^ 24 h; P=0.05 TLR4^loxP/loxP^ 48 h *vs* TLR4^loxP/loxP^ 24 h; n=5). Data are mean ± SEM.

Supplementary Figure 4 Analysis of neutrophil phagocytic activity. Two-way ANOVA analysis showed a significant effect of the time variable and the Bonferroni post-hoc showed a decrease in the phagocytic activity of neutrophils from TLR4^loxP/Lyz-cre^ mice (*P < 0.05, F[2, 19]=12.92; n=4-5).

Supplementary Figure 5 NETosis in the absence of TLR4. Percentage of NET-like events (H3Cit^+^/Elastase^+^) (n=4-6).

Supplementary Figure 6 ROS production by neutrophils in the absence of TLR4. **(A)** Post-ischemic analysis of ROS production (24 and 48 h after the stroke). **(B)** Two-way ANOVA analysis of the data showing a significative effect of time in both genotypes (P<0.05) and a significative difference between activated neutrophils of both genotypes at pre-surgery time point (*P < 0.05, F (1.028, 13.87) = 6.063; n=4-6).

## Abbreviations

BBB, blood brain barrier; CNS, central nervous system; DAMPS, damage-associated molecular patterns; LPS, lipopolysaccharide; MCAO, middle cerebral artery occlusion; MFI, mean fluorescent intensity; MPO, myeloperoxidase; MRI, magnetic resonance imaging; NET, neutrophil extracellular trap; PMA, Phorbol 12-myristate 13-acetate; RBC, red blood cells; ROS, reactive oxygen species; TLR4, Toll-Like Receptor 4; WBC, white blood cells; WT, wild type; ZT, Zeitgeber.
