# Supplementary figures and images for: Role of TLR4 in Neutrophil Dynamics and Functions: Contribution to Stroke Pathophysiology
Source: Front Immunol. 2021 Oct 21;12:757872. doi: 10.3389/fimmu.2021.757872 (PMC8566541; doi:10.3389/fimmu.2021.757872)

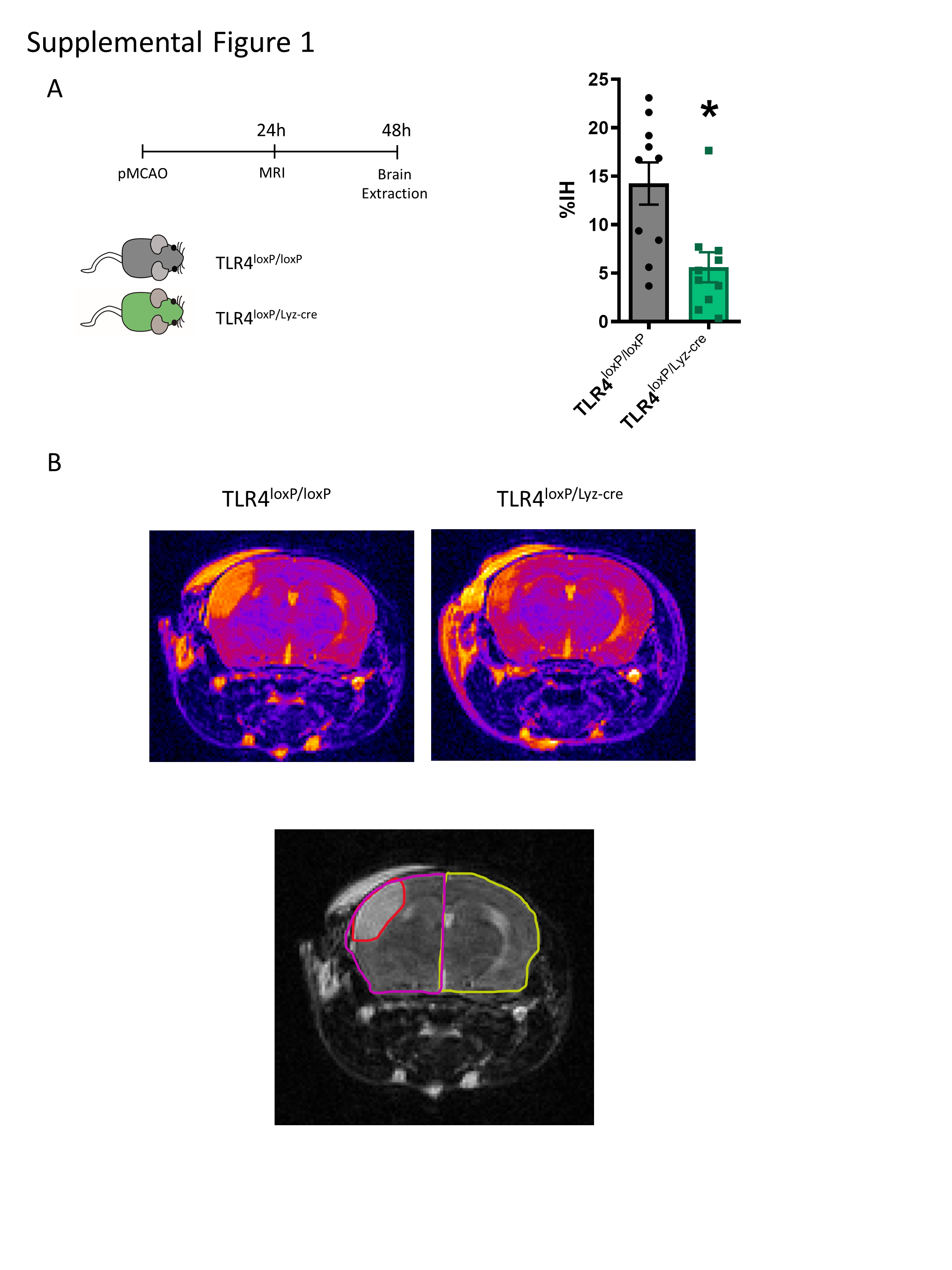

Supplement: Supplementary Figure 1 — Effect of specific myeloid TLR4 ablation on infarct volume. (A) TLR4loxP/loxP and TLR4loxP/Lyz-cre mice were subjected to MCAO; infarct volumes were determined by magnetic resonance imaging (MRI) 24 h after ischemic insult in both groups (n=10; *P < 0.05 vs TLR4loxP/loxP). (B) Representative images of MRI from both genotypes (top). Schematic representation of the measured areas from MRI images (Magenta: ipsilateral hemisphere; Yellow: contralateral hemisphere; Red: infarct). Data are mean ± SEM. [file Image_1.tif]

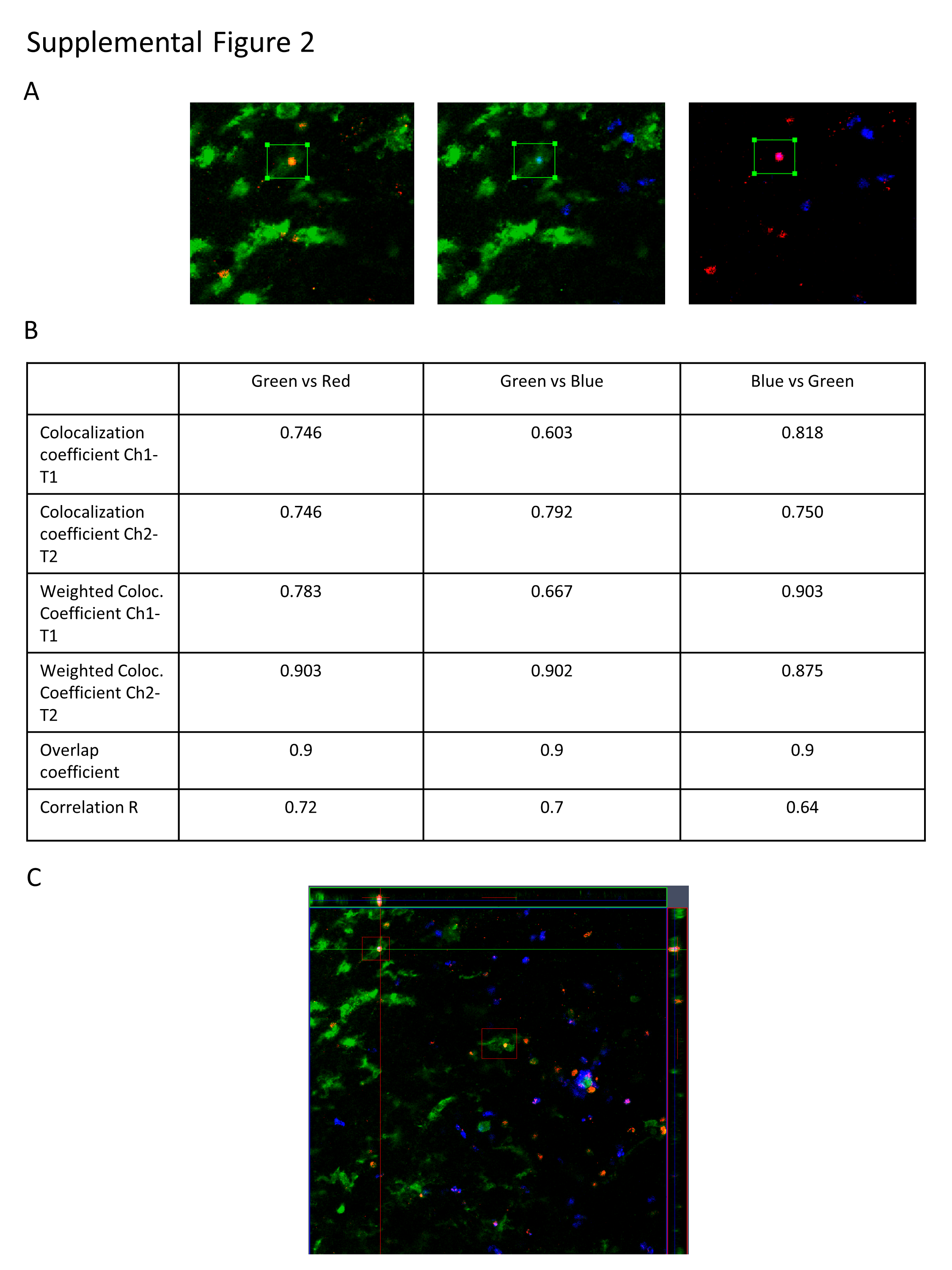

Supplement: Supplementary Figure 2 — Microglia engulfment colocalization analysis. (A) Representative images used for the colocalization analysis of the different channels two by two (green vs red, green vs blue and blue vs red). (B) Table containing the different values obtained with the colocalization analysis tool (Zen software, Zeiss). (C) Orthogonal projection of the representative microglia cell used to calculate the colocalization parameters (Green: Iba1, blue: NIMP-R14, red: Ym1). [file Image_2.tif]

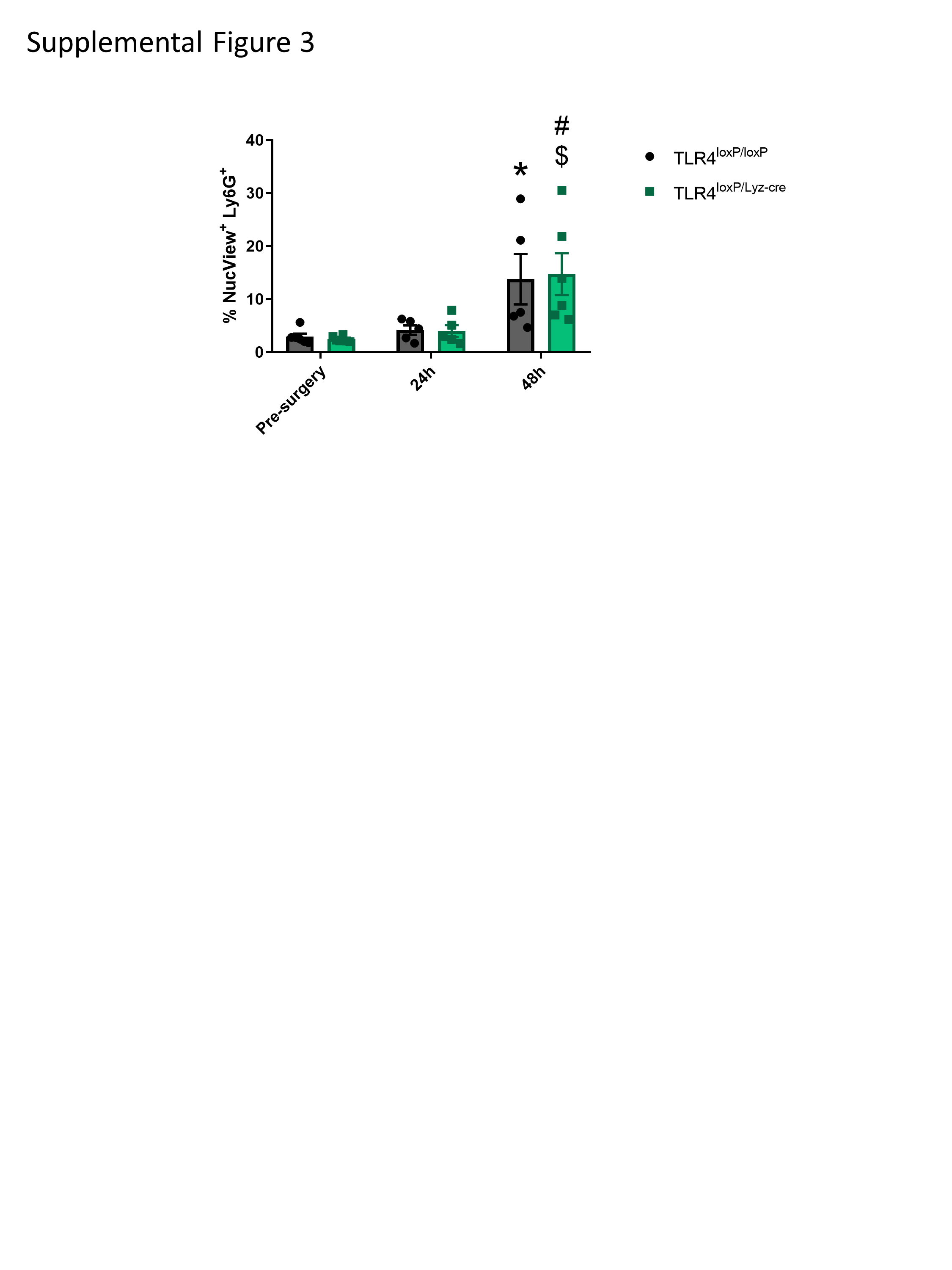

Supplement: Supplementary Figure 3 — Apoptosis assay in blood neutrophils. Two-way ANOVA analysis of the data showing a significative effect of time in both genotypes (Time: P<0.05, F[2, 10] 6.536; (*P < 0.05 vs TLR4loxP/loxP pre-surgery; #P < 0.05 vs TLR4loxP/Lyz-cre pre-surgery; $P < 0.05 vs TLR4loxP/Lyz-cre 24 h; P=0.05 TLR4loxP/loxP 48 h vs TLR4loxP/loxP 24 h; n=5). Data are mean ± SEM. [file Image_3.tif]

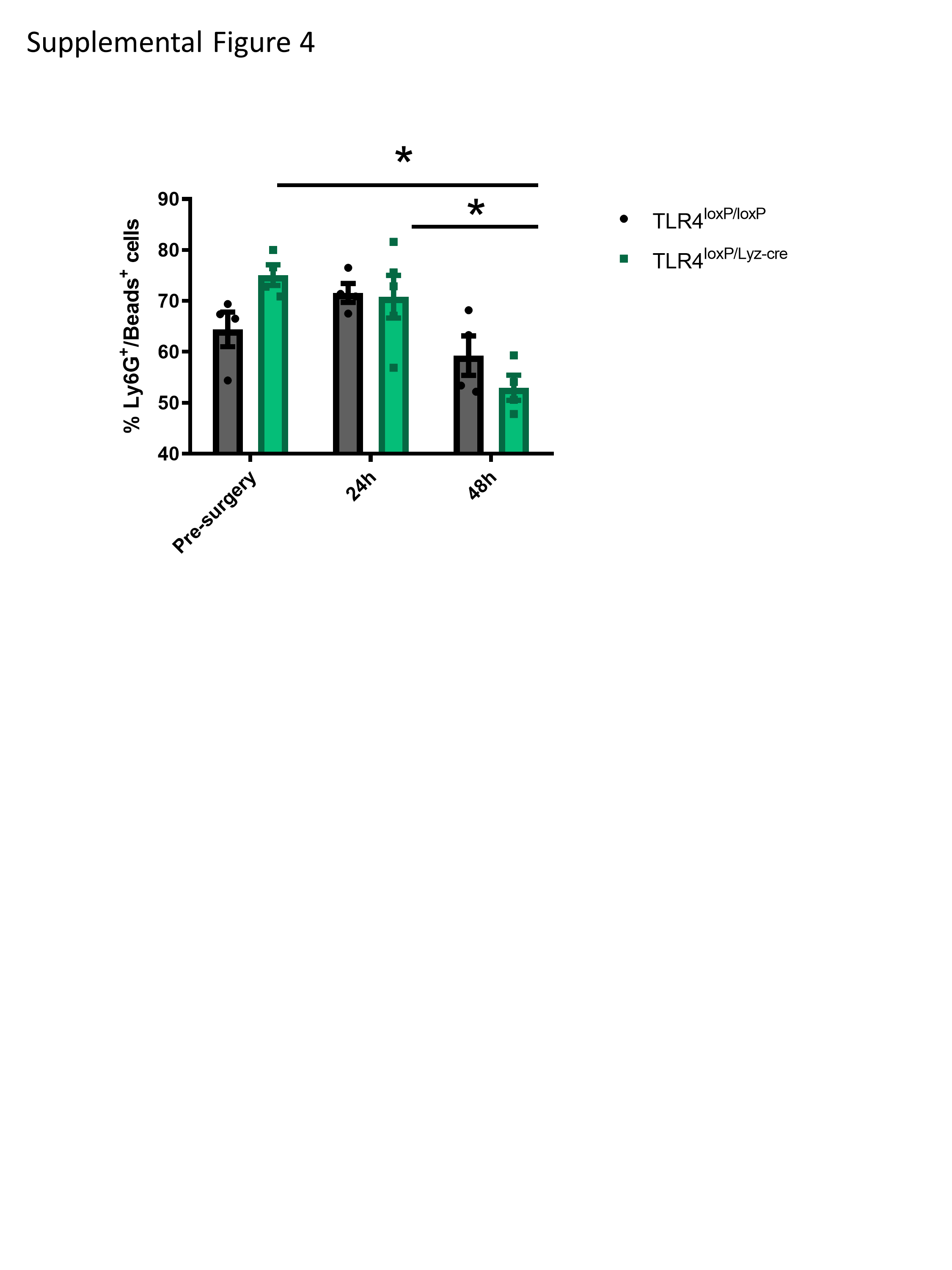

Supplement: Supplementary Figure 4 — Analysis of neutrophil phagocytic activity. Two-way ANOVA analysis showed a significant effect of the time variable and the Bonferroni post-hoc showed a decrease in the phagocytic activity of neutrophils from TLR4loxP/Lyz-cre mice (*P < 0.05, F[2, 19]=12.92; n=4-5). [file Image_4.tif]

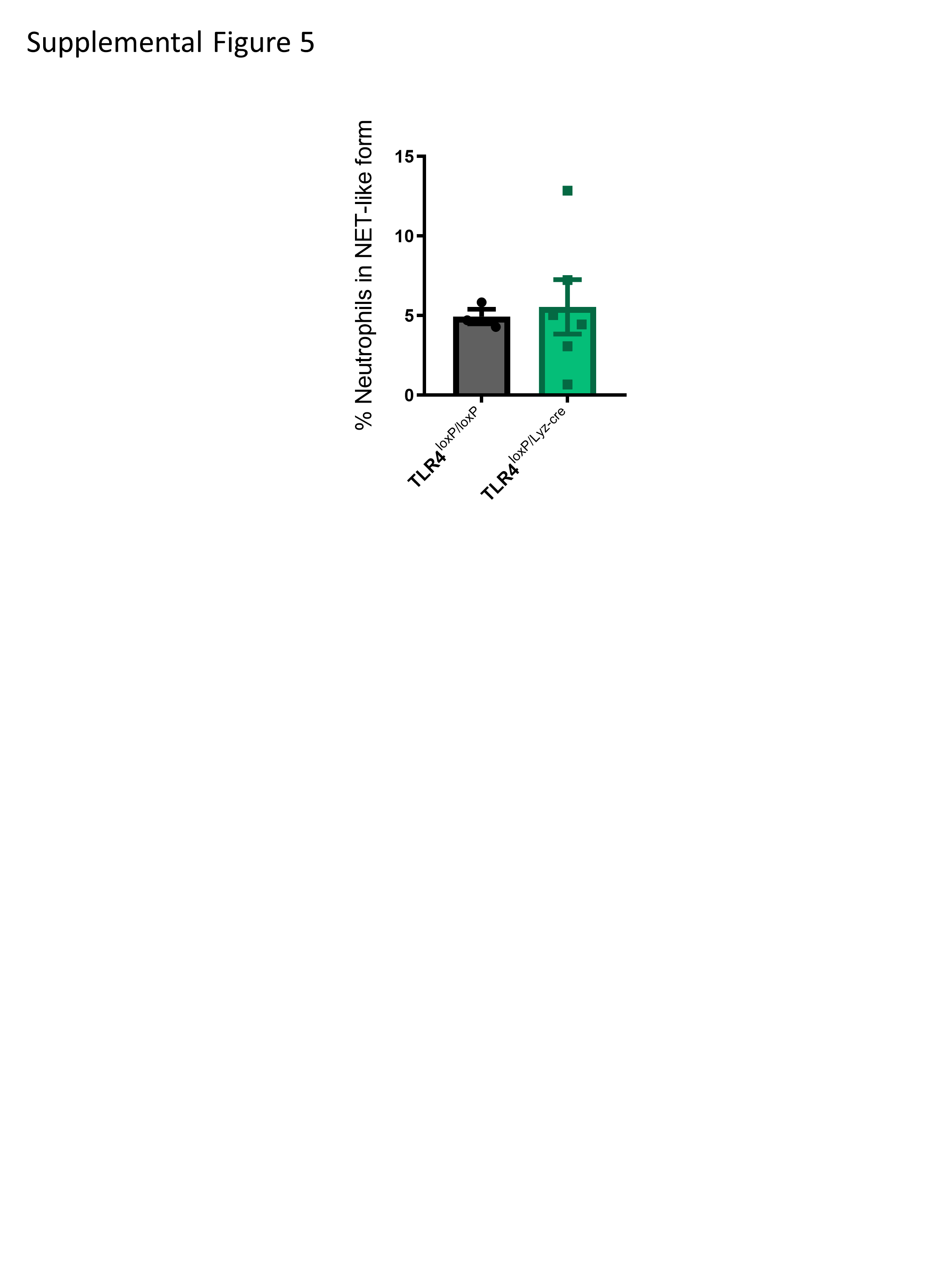

Supplement: Supplementary Figure 5 — NETosis in the absence of TLR4. Percentage of NET-like events (H3Cit+/Elastase+) (n=4-6). [file Image_5.tif]

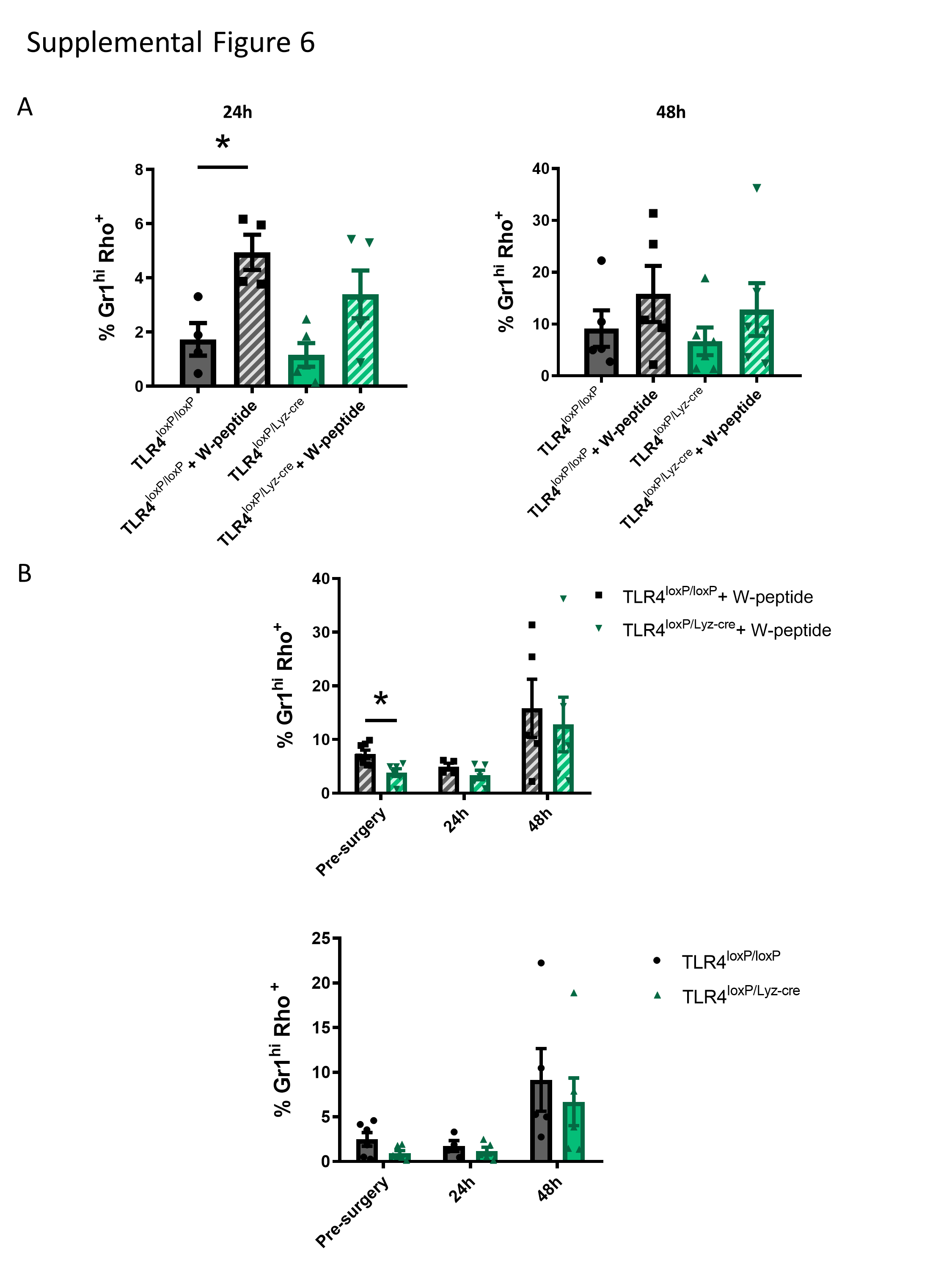

Supplement: Supplementary Figure 6 — ROS production by neutrophils in the absence of TLR4. (A) Post-ischemic analysis of ROS production (24 and 48 h after the stroke). (B) Two-way ANOVA analysis of the data showing a significative effect of time in both genotypes (P<0.05) and a significative difference between activated neutrophils of both genotypes at pre-surgery time point (*P < 0.05, F (1.028, 13.87) = 6.063; n=4-6). [file Image_6.tif]
